# Adapting Continuous Positive Airway Pressure Therapy to Where Patients Live: A Comparative Case Study

**DOI:** 10.7759/cureus.4078

**Published:** 2019-02-15

**Authors:** Edward J Miech, Dawn M Bravata, H. Klar Yaggi, Charles Austin, Lauren A Tobias, Jared Ferguson, Marianne S Matthias

**Affiliations:** 1 Internal Medicine, Roudebush VA Medical Center, Indianapolis, USA; 2 Internal Medicine, Yale University School of Medicine, New Haven, USA

**Keywords:** comparative case study, cpap, sleep apnea, adherence, home-based therapy, patient experience

## Abstract

Objectives

Continuous positive airway pressure (CPAP) therapy has been demonstrated to effectively reverse the abnormal physiology of sleep apnea and improve a variety of patient outcomes, yet helping patients adapt and adhere to CPAP has proven to be a challenging issue in the effective treatment of obstructive sleep apnea (OSA). As a home-based intervention trial, the “Sleep Apnea in Transient Ischemic Attack and Stroke: Reducing Cardiovascular Risk with Positive Airway Pressure” (“sleep tight”) study was uniquely positioned to capture and evaluate challenges faced by patients over time during their introduction to CPAP therapy.

Methods

A comparative case study design was used to better understand the process whereby patients adapted CPAP therapy in order to fit their own personal set of circumstances. Cases were identified from patients enrolled in the “enhanced intervention” group of the sleep tight trial.

Results

These comparative case studies illustrated how adherence to CPAP therapy is an adaptive process where personal context matters. The case studies also demonstrated how some patients overcame challenges and barriers by themselves to integrate CPAP therapy into their own lives, while others required help from study staff to overcome these barriers and some were never able to successfully adapt CPAP therapy in order to fit their personal contexts, despite study staffs’ best efforts.

Conclusions

A major opportunity to improve CPAP adherence appears to exist in placing greater emphasis on supporting patients in adapting CPAP therapy for “where they live.”

## Introduction

Continuous positive airway pressure (CPAP) therapy has been demonstrated to effectively reverse the abnormal physiology of sleep apnea and improve a variety of patient outcomes, yet helping patients adapt and adhere to CPAP has proven to be a challenging issue in the effective treatment of obstructive sleep apnea (OSA) [1]. Adherence rates vary across studies and across populations, but in general, adherence among adults has been reported in the 40% to 72% range [1-2]. A variety of educational, psychological, and technical approaches to improving CPAP adherence have been evaluated with mixed and generally modest effects [2]. As CPAP therapy inserts a brand-new element into patients’ bedrooms and bedtime routines, successful CPAP adherence can rely heavily on adapting CPAP therapy to “where patients live,” taking into consideration the specifics of personal context as well as the characteristics of individual patients [2]. 

As a home-based intervention trial, the “Sleep Apnea in Transient Ischemic Attack and Stroke: Reducing Cardiovascular Risk with Positive Airway Pressure” (“sleep tight”) study was uniquely positioned to capture and evaluate challenges faced by patients over time during their introduction to CPAP therapy. The sleep tight study was a randomized controlled trial that sought to evaluate the effectiveness of a diagnosis and treatment strategy for OSA among patients with a recent stroke or transient ischemic attack (TIA) on several domains of vascular risk. As part of the sleep tight protocol, patients in the intervention groups underwent polysomnography and received CPAP for up to one year if OSA was present [3].

Our objective in this study was to understand the process by which CPAP therapy was adapted in order to fit the personal context of patients after a diagnosis of OSA.

## Materials and methods

Overview of the sleep tight study

As described above, the sleep tight study was a randomized controlled trial seeking to evaluate the effect of CPAP therapy on vascular risk in patients with an acute ischemic stroke or TIA [3-4]. A total of 252 patients from six clinical sites in Connecticut and Indiana were randomly assigned to one of three groups: a usual care control group and two intervention groups. Patients in the intervention groups received polysomnography at home soon after their cerebrovascular event. A diagnosis of sleep apnea was based on an apnea-hypopnea Index (AHI) of five or greater [5]. Patients with OSA were offered CPAP for a minimum of six months and up to one-year post-enrollment. Intervention patients were randomly assigned to either the “standard intervention” or a multifaceted “enhanced intervention.” All participants provided written informed consent for participation in the study; this study received the Institutional Review Board (IRB) approval from the Yale University Institutional Review Board (1101007811) and the Indiana University Institutional Review Board (1105005279). This trial was registered with clinicaltrials.gov (NCT01446913).

The “standard intervention” patients received five in-person contacts and one telephone contact over the course of the up-to-12 month follow-up period. Research staff provided a standardized educational session about sleep apnea and CPAP, the results of the diagnostic sleep study and instruction regarding CPAP equipment during the initial in-person contact. Reviews of CPAP adherence data, discussions regarding symptom improvement and adverse effects, and encouragement of continued use were provided during in-person contacts.

Enhanced intervention of the sleep tight study

The “enhanced intervention” consisted of several components designed for more intense encouragement of CPAP adherence than is typically provided at sleep centers in routine clinical practice settings. The “enhanced intervention” protocol was based on the conceptual frameworks of the narrative model of patient decision-making and self-determination theory and consisted of intensive in-person patient contact during the first month of CPAP treatment and then telephone follow-up or in-person contacts for continuing support during the rest of the study period [6-8].

The protocol included targeted education, a customized behavioral adherence intervention, and increased CPAP support, with the aim of improving CPAP adherence over time. Targeted education included an introduction to the pathology of sleep apnea and its health consequences; a review of the patient’s sleep study results with a graphic image of their recorded sleep pattern illustrating their apneic events; instruction on the use of CPAP equipment and its role in treating sleep apnea including a discussion regarding the possible benefits of utilizing CPAP therapy (e.g. potential to reduce daytime sleepiness and fatigue, increased energy and enjoyment of daily activities). Study staff asked patients targeted questions to elicit their understanding of the information and sought to address any identified knowledge gaps. In the behavioral adherence component, study staff attempted to elicit motivating factors salient to individual patients as they initiated the use of CPAP, discussed potential challenges to CPAP use, and established goals and strategies for optimal adherence. The increased CPAP support in this trial consisted of inquiries regarding symptom relief and adverse effects as well as troubleshooting equipment needs at frequent intervals. Study staff members were specifically trained to develop individualized support plans that varied according to patient needs. Each visit also included a data download from the CPAP machine as an objective measure of the patient’s CPAP use, and this information was used to guide a dialogue about barriers and facilitators to CPAP use. Whenever possible, the same study staff member interacted with an individual patient in order to facilitate continuity of care and thereby maximize trust and rapport.

Each patient’s case was summarized in written form and discussed among an interdisciplinary team consisting of study staff familiar with the particulars of each patient, sleep physicians (HKY, LT), an expert in health-related communications (MM), and an expert in adult education (EJM). The goal of these discussions was to troubleshoot any problems related to that patient’s adherence and identify additional strategies tailored to that individual’s needs and circumstances that could potentially optimize adherence. As part of these conversations, we also reviewed the patients’ National Institutes of Health Stroke Scale (NIHSS), a measure of stroke severity where increasing scores indicate higher symptom severity, seeking to understand how stroke symptom severity might relate to CPAP use [9]. Suggestions generated in this forum were then brought back to the patient by study staff as they continually readdressed issues of adherence with individual patients.

Classification of CPAP adherence

CPAP adherence was measured in terms of the number of nights of any use and number of hours of use per night. Adherence was classified into three groups: “good” if usage was at least four hours per night for at least 70% of nights; “some” if the patient had used the device at least 10% of nights but less than threshold for “good”; and “none/poor” if the patient used the device less than 10% of nights [10].

Comparative case study approach

A comparative case study design was used to better understand the process whereby patients adapted CPAP therapy in order to fit their own personal contexts [11-12].“Personal context” in this study included the physical environment of patients’ homes and bedrooms; the mental constructs used by individual patients to represent how CPAP fit into their worlds; and the overall lives of patients, which included the impact of chance events as well as the impact of other developments related to family, significant others, work, and health. To assist with the challenge of successfully integrating CPAP into individual patient’s personal context, study team personnel visited patients in their homes to introduce CPAP, made follow-up home visits to provide ongoing support for CPAP adaptation and adherence, and offered regular support and troubleshooting advice over the telephone or in person.

Cases were identified from a subset of patients enrolled in the “enhanced intervention” group of the sleep tight trial. We explicitly sought to examine whether and how CPAP implementation was a developmental and adaptive process that unfolded over time, as opposed to a discrete event at a single point in time. The five individual cases in this analysis were selected to represent a wide range of adherence behaviors, attitudes toward CPAP, and a diversity of home situations. The sample size of five, representing 11% of the cases in the “enhanced intervention” group, was small enough to allow for in-depth analysis within and across cases in our comparative case study design.

## Results

A total of 56 patients in the “enhanced intervention” received polysomnography, and 45 (80%) had OSA. Among the patients with OSA, 18 (40%) had good CPAP use, 16 (36%) had some CPAP use, and 11 (24%) had poor or no CPAP use.

Case studies overview

Without exception, patients newly diagnosed with sleep apnea faced barriers, obstacles, and setbacks during the process of attempting to use CPAP; successful adherence relied heavily on how individual patients and study staff anticipated and responded to these emergent challenges and adapted CPAP therapy in order to fit personal contexts. 

Patient A

Patient A provided a striking visual example of personal adaptation during the CPAP implementation process. He was a college-educated, 64-year-old Asian male who experienced a stroke but had only minor deficits, with an NIHSS score of one. He reported chronic sleepiness, including falling asleep when driving or reading books. His initial AHI was 6.7 events per hour, consistent with mild sleep apnea.

**Figure 1:**
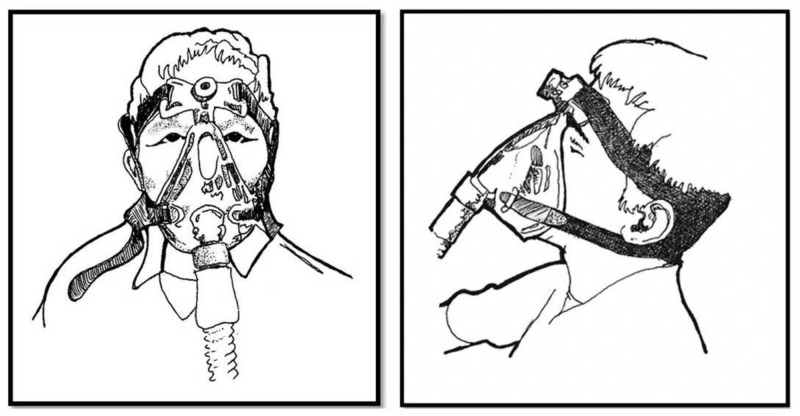
Comparison of original CPAP interface configuration (left panel) with modification of the CPAP interface designed and implemented by Patient A (right panel) CPAP: continuous positive airway pressure

Patient A started treatment with CPAP after his initial fitting visit, when he was instructed on the machine’s use and was fitted with a mask. Study staff identified a “salient positive” (any factor that was important to the patient that was expected to positively influence CPAP adherence) for Patient A as a desire to increase his daytime energy and reduce his daytime sleepiness. He did not report any negative concerns associated with CPAP therapy. He initially agreed to use a nasal mask but reported frustration with a sensation of low airflow. During an in-home follow-up visit in the first few weeks, Patient A requested a replacement mask that specifically fit over his nose and mouth. By the next week, he reported irritation from the strap that wrapped around the back of his head. He asked permission to modify the mask based on his own analysis of the interface and his background as an engineer. The study staff encouraged such modification and the patient developed a comprehensive physical adaptation with straps made with the help of kitchen shears and rolls of Velcro® (Figure 1) that allowed the CPAP mask to fit his face more comfortably and reduce the pressure of the straps on the back of his neck. In constructing his own modified interface with the CPAP mask, Patient A both figuratively and literally took ownership of the process of adapting CPAP therapy to fit his own personal context. By the end of the one-year study period, Patient A was using CPAP for four hours per night, consistent with “good” use.

Patient B

Patient B was an 83-year-old previously healthy white woman who lived with her husband, an attorney, in a new, well-furnished home. She was functionally independent, traveled frequently, and was socially active in local civic and religious organizations. Despite careful review of her medical records confirming that she had had a stroke, the participant was in denial about this diagnosis, claiming that it was nothing more than a dizzy spell and that doctors were never able to confirm that she had a stroke. During her baseline assessment, she had normal blood pressure, no cognitive deficits, an NIHSS score of 0, and reported daytime sleepiness and fatigue. 

She was pleasant and polite when scheduling her appointments, but was often anxious about the in-home visits and would frequently call study staff to confirm a visit if the research staff was running as little as five minutes late. In addition, Patient B made it clear that she had other priorities that took precedence over the diagnosis and treatment of her OSA. For instance, during her initially scheduled in-home polysomnographic evaluation, Patient B was reluctant to have the sleep study because she had just been to the hairdresser and feared wearing the equipment would ruin her newly-coiffed hair. She, therefore, requested that the study is rescheduled to a night before her next scheduled hair appointment, and this was accommodated by study staff.

With an AHI of 46, the sleep study revealed obstructive sleep apnea in the severe range and CPAP was presented to her as a treatment. Salient positives that could potentially promote CPAP adherence included her pre-existing understanding of CPAP resulting from a family member’s prior use of the machine and her desire to decrease her current daytime sleepiness. Salient negatives that could serve as potential barriers included her concern for the condition of her hair and the potential for discomfort from the pressure of the mask. She seemed to understand the benefits of using CPAP; however, she voiced a guarded willingness to use the device because she continued to question her stroke diagnosis and how CPAP therapy would fit into her cerebrovascular treatment regimen.

Patient B’s first few months of CPAP were successful: she wore the mask 85% of nights, for greater than four hours each night. However, during this timeframe, research staff received many calls from the patient, which resulted in several unscheduled visits to provide emotional and technical support. The staff members adjusted pressures, re-educated her about the use of the ramp button to manage the machine’s pressure and addressed her concerns about nosebleeds, nasal dryness, and nasal bridge soreness. During many visits, the participant revealed forgetfulness and confusion related to the use of the machine; during one visit, staff discovered she was wearing the nasal mask upside-down. 

Between her fourth and fifth months in the study, she did not use CPAP. At her sixth month of participation, Patient B continued to complain of her inability to sleep for more than a few hours because of a sensation that the air pressure of the machine was too high, despite having lowered the maximum pressure provided by the CPAP machine. Unable to lower the pressure any further without compromising effectiveness, and based on the team’s review of the case, study staff informed Patient B that using the machine even a few hours a night would be better than not using it at all, and recommended she wears the mask until waking up the first time and then remove it for the remainder of the night. An examination of the temporal relationship between the patient’s use of the CPAP machine and study staff contacts during the course of the study indicated that adaptation suggestions provided by the study staff helped her to adapt CPAP use in light of her personal circumstances. By the end of the one-year study period, Patient B was using CPAP for between four and five hours per night, consistent with “good” use.

Patient C

Patient C was a 47-year-old white female with a recent transient ischemic attack (TIA) and an AHI of 10, consistent with mild sleep apnea. She described her husband as very supportive of her efforts to use CPAP. The study staff identified several salient positives, leading to the judgment that she had a high probability of successfully using CPAP. First, she was a nurse and had a greater understanding of the complications of OSA and the benefits of CPAP than the typical layperson. In addition, she believed that her husband might have OSA, and articulated a goal of demonstrating good CPAP adherence herself to “set a good example” that might encourage him to seek and adhere to treatment. Finally, she indicated that she was feeling extremely tired, pointed to bags under her eyes, and perceived CPAP as a means to improve her energy. The one potential negative was that she was not interested in having anything cover her face. She was highly motivated and told study staff that she was going to be their “best user”.

During her first week with CPAP, she was a very good user of the device (at least four hours per night) and she described feeling quite positive about CPAP. However, as she continued to use it, she began experiencing issues with abdominal bloating that caused her to discontinue use. She had a vacation planned, and, although she originally reported an intention to take the machine with her, aerophagia ultimately resulted in her decision to leave the device at home. After her vacation, study staff addressed the issue by changing from a full-face mask interface to a nasal cannula.

Initially, these changes resulted in improved use, but she then experienced the sensation of increased pressure, which caused her to awaken in the middle of the night. She expressed to study staff a frustration that she was not the “best user”. Study staff responded by re-educating her about the ramp button to reduce the pressure and offered her encouragement. Her CPAP adherence improved, but she still experienced difficulties with nocturnal awakenings and a sensation of uncomfortably high pressures. By the end of her participation in the study, she was using CPAP for at least four hours per night for three-quarters of nights but was still “ashamed” that she was not a “better” user.

Patient D

Patient D was a 40-year-old African American woman whose living situation involved unstable and economically impoverished conditions. She had not finished high school, and her stroke deficits compounded her inability to find employment. She lived with three daughters, various house guests, and grandchildren; they resided in three different locations during the 12-month study period.

Her home during the initial sleep study was a two-story rental house located in a high-crime neighborhood. The living room had two sofas and a love seat. During the first staff visit, Patient D’s daughter was asleep on one of the sofas and a man was asleep on another. The patient slept and spent most of her waking hours on the third. The hardwood floors were covered in cigarette ash, fast food bags and cups, and plastic bags filled with clothing. The window in the living room was broken and covered with shredded plastic that allowed outside air to blow into the room.

The patient had a history of noncompliance with prescribed medications. For example, study staff learned that she had not been taking her hypertension medications, reportedly due to cost. As a consequence, Patient D often had elevated blood pressure during her participation in the study. 

Patient D’s initial sleep study was difficult to perform due to her inability to sit for long periods of time. The patient removed the equipment before bedtime, resulting in a failed study. However, she was willing to try again and the second time the study was successfully completed with the exception that she refused to allow the placement of some of the leads and pulled off others before falling sleep. Despite these problems, her AHI was found to be 10, consistent with mild sleep apnea, and she expressed willingness to try CPAP therapy. A salient positive factor promoting potential CPAP adherence was that she demonstrated a good understanding of the benefits and struggles of CPAP use, and over the course of the next few weeks she began to use the CPAP machine. Unfortunately, it was at this point that her electricity was turned off, making it impossible for her to use the device for nearly a month. 

After a month, she tried to use the CPAP again but struggled with mask discomfort. In addition, she reported a frequent, yet sporadic on/off malfunction of the device. Over the remainder of the patient’s participation in the study, research staff re-educated her about pressure settings, encouraged use, offered support, wrote two letters to the electric company in an effort to keep the utility connected, cleaned the device and eventually replaced it due to malfunction. One month after the end of the study, the patient requested a third letter to the electric company confirming her use of the machine and resulting medical need for electricity. At the conclusion of the study, the participant’s utilization was infrequent, illustrating how conditions related to personal context can impede successful adaptation to CPAP therapy.

Patient E

Patient E was a 47-year-old African-American woman had a history of TIA, hypertension, hyperlipidemia, depression, morbid obesity, myocardial infarction, and prior tobacco use. She was enrolled in the study after a left middle cerebral artery stroke that resulted in right upper and lower extremity weakness. Based upon the initial assessment, staff predicted that she had a high probability of successfully using CPAP. Salient positives included the ability to identify several motivators for CPAP adherence including significant tiredness that limited her ability to participate in daily activities such as shopping or walking. She had an AHI of 16, consistent with moderately severe OSA. 

She was immediately optimistic about using CPAP and reported no initial barriers to use or adverse effects within the first week of therapy. She reported a reduction in sleepiness after just the first night’s use. On subsequent visits, she frequently demonstrated enthusiasm about CPAP (“I’m so glad you found this [OSA]”) and expressed her gratitude to study staff on each visit. She used CPAP for over four hours per night. 

She stated that the only time she did not use her CPAP machine was when she and her husband were sexually active, approximately once weekly. An initial barrier to use was her removal of the mask when she awoke in the middle of the night to use the bathroom, but staff provided education, encouraging her to leave the mask on her face and simply disconnect the tubing in order to increase the likelihood of continuing CPAP for the remainder of the night.

This patient faced a number of challenges: She was limited in her mobility and walked with a cane. At one point during the study, she fell into a bathtub and needed assistance getting up from the fall. Eleven months after CPAP delivery, she reported a bedbug infestation requiring her to move into her sister’s home during fumigation, but she resumed CPAP use once home again. Despite these challenges, her end-use was between four and five hours per night, consistent with “good” use, indicating that her successful adaption of CPAP could overcome chance adverse events. 

## Discussion

Patients who are diagnosed with OSA are typically placed on a CPAP treatment plan, often with little follow-up until several months later. During the process of beginning to use CPAP, however, patients must go through a period of adaptation, and their experiences during this early period can set the stage for success or failure to adhere to treatment in the long term. The sleep tight study employed an enhanced intervention in which study staff contacted patients frequently during this initial period of adjustment to CPAP, providing a unique opportunity both to understand how patients navigate this adjustment period and adapt CPAP therapy in order to better fit their personal contexts. The study staff was actively involved in patients’ early experiences with CPAP, which other work has suggested may be key in predicting later CPAP use [13]. Some patients were able to navigate this adaptation process independently, with little or no help from staff, such as patient A, who modified his own interface so that he could wear it more comfortably. Others welcomed and required ongoing support from study staff, who helped patients adapt CPAP therapy to fit their own set of circumstances through multiple in-home visits and regular support over the phone. 

These comparative case studies illustrated how adherence to CPAP therapy is an adaptive process in which personal context has critical importance. This finding implies that there appears to be a major opportunity to improve CPAP adherence by placing greater emphasis on our support of patients as they adapt CPAP therapy to their own personal contexts. As with the initiation of many medical treatments (e.g., antihypertensive medications), physicians are likely to guide patient encounters toward a discussion of the medical benefits of using therapy and/or the adverse outcomes that may result from failure to comply with therapy. For example, in our experience, most sleep physicians’ initial encounters with patients newly diagnosed with OSA focus on the improvements in daytime sleepiness and cognitive functioning that may result from good CPAP adherence, and the adverse cerebrovascular, cardiovascular, and metabolic consequences of untreated OSA. However, the simple provision of CPAP and the communication of evidence related to OSA may be inadequate for successful CPAP adaptation. CPAP adaption is a process that unfolds over time, and our current single-visit model may be insufficient to drive consistent use [14]. Moreover, it may be particularly important to support patients with stroke or TIA, such as those in the Sleep Tight study, as they integrate CPAP therapy into their own lives and routines. These patients may be less symptomatic for sleepiness and thus fail to seek medical help specifically for sleep-related problems [15]. This lack of specific sleep-related symptoms might negatively affect motivation to persevere with CPAP and adapt it to the personal context.

Prior work demonstrated that there appears to be a continuum that describes the developmental and adaptive process that individual patients experience when implementing CPAP [14]. Patients moved gradually along this continuum over time. The present study extended that set of findings by illustrating how adaptation played a role in patient adherence. The current study also demonstrated how some patients overcame challenges and barriers by themselves to integrate CPAP therapy into their own lives, while others required help from study staff to overcome these barriers. Others were never able to successfully adapt CPAP therapy in order to fit their personal contexts, despite study staffs’ best efforts. 

This study has several important limitations. The study participants were tested for OSA and not because of sleep-related complaints but because they had suffered a stroke or TIA. Furthermore, the sample in this study was selected to represent a wide range of adherence behaviors, attitudes toward CPAP, and different home situations. As a result, generalizability to the larger population of patients with OSA may not be possible.

## Conclusions

This study used a comparative case-study methodology to provide exemplars of different patient experiences with CPAP and provided important insights into the process of adapting CPAP therapy to fit personal contexts and suggest important directions for further research. Specifically, future work should evaluate different strategies to assist patients in adapting CPAP into their lives and routines, including developing personalized approaches that account for patients’ home sleeping situations and personal motivators of CPAP use. In addition, this comparative case study indicated that the current widespread practice of prescribing CPAP without follow-up for weeks to months may be inadequate. Individualized and frequent contacts from outside staff can help troubleshoot several issues and facilitate adjustments that help patients successfully adapt CPAP within their own personal circumstances. Finally, this study illustrated how CPAP therapy can be seen as a “lifestyle modification,” where successful adherence requires new patterns of behavior and physical, psychological, and cognitive adaptations. Given the ongoing challenges associated with CPAP adherence, helping patients adapt CPAP therapy to where they live represents a major opportunity to improve CPAP adherence and improve OSA-related outcomes.
